# Towards a fundamental safe theory of composite Higgs and dark matter

**DOI:** 10.1140/epjc/s10052-020-08648-7

**Published:** 2020-11-24

**Authors:** Giacomo Cacciapaglia, Teng Ma, Shahram Vatani, Yongcheng Wu

**Affiliations:** 1 Université de Lyon, Univ. Claude Bernard Lyon 1, CNRS/IN2P3, IP2I Lyon, UMR 5822, 69622 Villeurbanne, France; 2 CAS Key Laboratory of Theoretical Physics, Institute of Theoretical Physics, Chinese Academy of Sciences, Beijing, 100190 China; 3 Physics Department, Technion-Israel Institute of Technology, 3200003 Haifa, Israel; 4 Ottawa-Carleton Institute for Physics, Carleton University, 1125 Colonel By Drive, Ottawa, ON K1S 5B6 Canada

## Abstract

We present a novel paradigm that allows to define a composite theory at the electroweak scale that is well defined all the way up to any energy by means of safety in the UV. The theory flows from a complete UV fixed point to an IR fixed point for the strong dynamics (which gives the desired walking) before generating a mass gap at the TeV scale. We discuss two models featuring a composite Higgs, Dark Matter and partial compositeness for all SM fermions. The UV theories can also be embedded in a Pati–Salam partial unification, thus removing the instability generated by the $\text{ U }(1)$ running. Finally, we find a Dark Matter candidate still allowed at masses of 260 GeV, or 1.5–2 TeV, where the latter mass range will be covered by next generation direct detection experiments.

## Introduction

The ultra-violet (UV) behaviour is crucial for a quantum field theory (QFT) to be predictive and fundamental up to high scales [1, 2]. The presence of fixed points in the renormalisation group evolution of gauge and non-gauge couplings plays a central role in this. The prime example is quantum chromo-dynamics (QCD), which features a free fixed point where the gauge coupling vanishes in the UV [3, 4]. The possible existence of an interacting fixed point has been first proposed by S. Weinberg in the context of quantum gravity [5], but prematurely discarded for renormalisable QFTs. Until, Sannino and Litim [6] found a first example of perturbative interacting UV fixed point in a theory with scalars and gauge-Yukawa couplings. Safe pure gauge theories with fermions have been obtained by employing resummation techniques for large number of fermionic flavours [7–9]. Recent progress can be found in Refs. [10–14]. For a simple gauge group with $N_f$ fermions in the representation $r_f$, the resummed beta function reads:

1 $$\begin{aligned} \frac{\partial \ln K}{\partial \ln \mu } \equiv \beta (K) = \frac{2 K}{3} \left[ 1 + \sum _{n=1}^\infty \frac{1}{N_f^n} B^{(n)} \right] , \end{aligned}$$

where $K = N_f T(r_f) \alpha /\pi$. The presence of a UV fixed point is hinted by the fact that the first term of the expansion $B^{(1)}$ has a negative pole at a finite value of *K* [10]. Note that only the first term in the large $N_f$ expansion is known.^1^ The analysis based on resummation has been recently challenged in Ref. [16] but without conclusively disproving the existence of a fixed point [17], while studies on the lattice are still inconclusive [18]. In this work we will assume that the presence of a pole in the large $N_f$ expansion is a sign of a genuine UV fixed point.

The least attractive feature of this class of asymptotically safe theories is the need for a large multiplicity of fermion matter fields, as it can be seen in the attempts to build a safe extension of the Standard Model [19, 20]. Whilst this possibility is not ruled out experimentally, postulating the presence of tens of new massive fermions at the multi-TeV scale for the sole purpose of changing the UV behaviour of the theory contradicts the principle of minimality.^2^ In this work we want to point out a class of theories where the presence of large multiplicities of heavy fermions is required for another crucial reason: the generation of masses for all Standard Model fermions in models of composite Higgs with partial compositeness. In these models, minimality requires that there exists one composite operator for each chiral fermionic field in the Standard Model. To generate such a spectrum of operators, an underlying theory needs to contain a large number of preons, the fundamental degrees of freedom that constitute the composite objects. Furthermore, considerations related to the hierarchy problem lead to postulating fermionic preons.

A classification of underlying theories based on gauge-fermion interactions can be found in Ref. [22]. The main constraint on this model building effort is precisely the requirement that the theory shall remain confining at low energies [23]. This limits the number of underlying fermions to the ones responsible for giving mass to the top quark only. This problem is absent in theories with scalar fields [24] at the price of reintroducing the hierarchy problem related to elementary scalar masses. In any case, these theories remain underlying descriptions of the composite Higgs dynamics of top partial compositeness, but far from being true UV completions. In fact, the origin of the light fermion masses as well as the source for the couplings generating the partial compositeness remain absent. Our goal is therefore to take a decisive step towards addressing these issues and being able to construct a genuine UV complete theory that can be trusted at arbitrarily high energies, at least up to the Planck scale. In this perspective, providing a Dark Matter candidate becomes a key ingredient.

In this work we present a new paradigm that allows to define composite Higgs models with underlying fermions up to arbitrary high energies. The large number of fermions needed to give mass to all standard quarks and leptons drives the theory to a complete UV interacting fixed point. The fermions associated with the two light generations are supposed to have a large mass, thus explaining the lightness of their partners compared to the electroweak scale. Once integrated out, the remaining degrees of freedom drive the confining gauge interaction towards an Infra-Red (IR) fixed point [25]. The resulting conformal window, similar in nature to walking Technicolor [26, 27], allows to further split the scale of the heavy fermions where flavour effects also arise, from the condensation and electroweak scales. The exit from the IR fixed point can be driven by integrating out a subset of the remaining light fermions, leaving one of the models of Ref. [22] at low scale. In this framework, fundamental scalar fields can also be added in a natural way,^3^ as long as their masses are close to the mass of the heaviest fermions [28], and they can be responsible for generating the needed four-fermion interactions. The low-energy flavour mixing of the SM can therefore be traced back to high-scale Yukawa couplings of scalars that, as we will show, are charged under the confining strong interactions. A Dark Matter candidate can be easily included in this class of theories [29–31]. The new scenario we discuss here, therefore, allows to define composite models that can in principle be as predictive as supersymmetric extensions of the Standard Model.

This paper is organised as follows: after introducing the general set-up in Sect. 2, we describe in Sect. 3 how two underlying models of top partial compositeness with Dark Matter can be extended to UV safety. In Sect. 4 we introduce the scalar sector at high energy, responsible for generating the flavour couplings. In Sect. 5 we embed the two models in a Pati–Salam unification framework, thus eliminating the $\text{ U }(1)$ problem. Finally, in Sect. 6 we analyse the phenomenology of the Dark Matter candidate, before offering our conclusions in Sect. 7.

## Choosing the model

**Table 1:** Minimal cosets with a pNGB Higgs doublet arising from an underlying gauge-fermion theory. The fourth column shows the $\text{ SU }(2)_L$ *irrep*, with the hypercharge as subscript. The last two columns show some properties of the explicit models, with the nomenclature M1-M12 from Ref. [32], and MV being the model from Ref. [33]

$\psi$ irrep	Coset	pNGBs	pNGB EW charges	Models	${\mathcal {G}}_\mathrm{HC}$
Pseudo-real	$\text{ SU }(4)/\text{Sp }(4)$	5	$2_{\pm 1/2} \oplus 1_0$	M8-M9	$\text{ Sp }(4)$, $\text{ SO }(11)$
Real	$\text{ SU }(5)/\text{SO }(5)$	14	$2_{\pm 1/2} \oplus 3_{\pm 1} \oplus 3_0 \oplus 1_0$	M1-M7	$\text{ SU }(4)$, $\text{ Sp }(4)$, $\text{ SO }(7)$, $\text{ SO }(9)$, $\text{ SO }(10)$
Complex	$\text{ SU }(4)^2/\text{SU }(4)$	15	$2 \times 2_{\pm 1/2} \oplus 3_0$	M10-M12	$\text{ SO }(10)$, $\text{ SU }(4)$, $\text{ SU }(5)$
			$\oplus 1_{\pm 1} \oplus 2 \times 1_0$	MV	$\text{ SU }(3)$

To define an underlying theory for composite Higgs models, we need to specify a confining hyper-colour (HC) gauge symmetry, ${\mathcal {G}}_\mathrm{HC}$, and the irreducible representation (*irrep*) of the underlying fermions, $\psi _i$. Furthermore, the electroweak (EW) quantum numbers of the $\psi _i$ should be suitably chosen such that a Higgs doublet arises as a pseudo-Nambu Goldstone boson (pNGB) after confinement and chiral symmetry breaking. The partial compositeness paradigm imposes a strong additional requirement: the presence of spin-1/2 bound states that mix with the standard fermions.

Bound states of three $\psi$’s are possible for the fundamental *irrep* of ${\mathcal {G}}_\mathrm{HC} = SU(3)$ [33], as long as some of the fermions also carry QCD charges in addition to the EW ones. Another possibility, proposed in Ref. [22], is to sequester QCD charges to a second class of underlying fermions, $\chi _j$, transforming under a different ${\mathcal {G}}_\mathrm{HC}$ *irrep*. The benefit of this choice is that the breaking of the EW symmetry via vacuum misalignment in the $\psi$-sector is decoupled from QCD, which shall not be broken. Furthermore, the spin-1/2 bound states that enter partial compositeness arise as chimera baryons [34] made of both species of fermions, in the two alternative forms

2 $$\begin{aligned} {\mathcal {B}} = \langle \psi \psi \chi \rangle \quad \text{ or } \quad \langle \psi \chi \chi \rangle . \end{aligned}$$

For each HC gauge group, the multiplicity of fermions, and their *irreps*, are limited by the requirement that the theory remains asymptotically free, i.e. it confines at low energy, and outside the IR conformal window [35, 36], i.e. a mass gap is generated at low energy. Furthermore, the minimal number of $\psi$’s is given by the requirement of having a Higgs doublet in the coset, while the minimal $\chi$ sector needs to contain a QCD colour triplet and an anti-triplet in order to generate – at least – the top mass. This leaves only 12 feasible models [23] with minimal Higgs cosets, which we denote M1 to M12, following Ref. [32]. The low energy models we consider here, and their key features, are listed in Table 1.

To define a genuine UV completion for these models, the issue of Dark Matter cannot be avoided. The simplest possibility is that one of the additional pNGBs may be stable. The minimal case is offered by the coset $\text{ SU }(4)_L \times \text{ SU }(4)_R/\text{SU }(4)$ [29, 37], which can be obtained in models M10-12, and the models of Refs. [24, 33]. In fact, the other two minimal cosets do not feature a stable pNGB because of the Wess-Zumino-Witten topological term. They could feature a DM candidate only if extended. As it was shown in Ref. [37], in the EW sector there is a unique ${\mathbb {Z}}_2$ parity that is conserved by the fermion condensate (if custodial symmetry is preserved) and by the EW gauging, as well as being anomaly free: it is defined in terms of charge conjugation in the $\psi$ sector plus a flavour rotation in the $\text{ SU }(4)$ flavour space. If the top couplings also respect this parity, the pNGB spectrum will contain several odd scalars, in particular a doublet and a triplet of $\text{ SU }(2)_L$ plus a neutral and a charged singlet. Such states mix, and the lightest neutral one plays the role of Dark Matter candidate (see Ref. [37] for more details on the pNGB structure).

To extend the Dark ${\mathbb {Z}}_2$ parity in the case of partial compositeness, we need to make sure that the composite operator ${\mathcal {B}}$ that mixes with the top has well-defined transformation properties and contains even states with the same quantum numbers as the top quark fields. As the Dark parity contains charge conjugation in the $\psi$-sector (but not $\chi$), it is crucial that the bound state contains two $\psi$’s: this simple fact rules out the case with HC-charged scalars of Ref. [24]. Furthermore, the $\psi$-bilinear in ${\mathcal {B}}$ needs to be in a real *irrep* of ${\mathcal {G}}_\mathrm{HC}$ (ruling out M12 and the model of Ref. [33]). We are therefore left with the models M10 and M11, based on $\text{ SO }(10)_\mathrm{HC}$ and $\text{ SO }(6)_\mathrm{HC} \equiv \text{ SU }(4)_\mathrm{HC}$ respectively, with $\psi _i$ in the spinorial ($\mathbf{Sp}$) *irrep* and $\chi _j$ in the fundamental ($\mathbf{F}$). For the top partners, there remains two choices that preserve the Dark parity: case a) a bi-fundamental of $\text{ SU }(4)_L\times \text{ SU }(4)_R$, which decomposes into a symmetric and an anti-symmetric of the unbroken $\text{ SU }(4)$; case b) a pair of symmetric *irreps*. As we will see in Sect. 6, only case a) leads to a feasible low energy model.

## A fundamental theory with UV safety and dark matter

In the following, we will focus on M10 and M11: they only differ in the HC group, $\text{ SO }(10)_\mathrm{HC}$ versus $\text{ SO }(6)_\mathrm{HC}$. The low-energy fermion content [22] consists of 4 EW-charged $\psi$’s and a QCD-coloured triplet of $\chi$’s, as shown in the upper block of Table 2. These fermions characterise the composite states below the condensation scale $\Lambda _\mathrm{HC}$, including the Higgs, the Dark Matter candidate and the top partners.

To complete the model, we will extend it by adding one appropriate $\chi$ for each standard fermion that acquires mass via the Higgs mechanism, as shown in the remaining two blocks of Table 2. We add the partners for the bottom quark and tau lepton at a scale close to $\Lambda _\mathrm{HC}$, *i.e.* 5 additional $\chi$-flavours. This is enough to push the theory into the conformal window (more details in Appendix A): right above the condensation scale, therefore, the strong sector flows into a conformal phase where the gauge coupling remains strong and slowly walking. This phase may ensure that the operators that mix to the light generations acquire a largish anomalous dimension, allowing to sufficiently decouple the scale where they are introduced. The $\chi$ fermions associated to the light generations are, in fact, introduced at a scale $\Lambda _\mathrm{Fl} \gg \Lambda _\mathrm{HC}$, where flavour effects are also generated. Above $\Lambda _\mathrm{Fl}$, the number of fermions is such that the running of all gauge couplings are not asymptotically free any more. The lepton partners, $\chi _l^{i}$, are chosen to be doublets of $\text{ SU }(2)_L$ for two reasons: on the one hand, their presence will assure that the $\text{ SU }(2)_L$ gauge coupling also runs into safety; on the other hand, the quantum numbers are such that chimera baryons containing $\chi _l^{i}$ also feature a neutral singlet, i.e. right-handed neutrinos, thus allowing to generate neutrino masses. We will first study how the gauge couplings of these theories may flow to a UV safe fixed point.

Our set up differs from the ones considered in the literature (see Ref. [10]) in the fact that we have different sets of fermions participating to the running of the four gauge couplings. Furthermore, for the $\text{ SO }({\mathcal {N}})$ group, there are two different *irreps* that need to be taken into account [38]. For these reasons, we define the large-$N_f$ gauge couplings as follows:

3 $$\begin{aligned} K_i \equiv N_i \frac{\alpha _i}{\pi }, N_i = \sum _f n_f T (r_f); \end{aligned}$$

with $i = 1,2,3,{\mathcal {N}}$ labelling the four gauge groups (for $\text{ U }(1)$, replace $T(r_f) \rightarrow Y_f^2$). As there are many fermions in different *irreps* of the gauge groups, in our case we cannot define a unique $N_f$ valid for all gauge coupling running, but rather we need to define a different multiplicity $N_i$ for each group. We will assume that formally they are all of the same order. For the extended models in Table 2, we find the multiplicity factors listed in Table 3. For the running of the $\text{ SO }({\mathcal {N}})$ gauge coupling, as there are two different *irreps* that contribute, we follow the results in Ref. [38]:

4 $$\begin{aligned} \frac{B^{(1)}_i}{N_f}= & {} \frac{C_2 (G_i)}{N_i} \left( - \frac{11}{4} + G_1 (K_i) \right) \nonumber \\&+ \sum _{j=1}^{{\mathcal {N}}} \frac{c_{i,j}}{N_j} F_1 (K_j), \end{aligned}$$

where the functions $F_1$ and $G_1$ are defined in 1, and $C_2 (G_i)$ is the Casimir of the adjoint of the *i*th gauge group (for the abelian case, $C_2 (G_1) = 0$). Note that the $-11/4$ term corresponds to the one loop contribution of gauge bosons. The coefficients $c_{i,j}$ are all positive, and their values can be found in Appendix B.

**Fig. 1:**
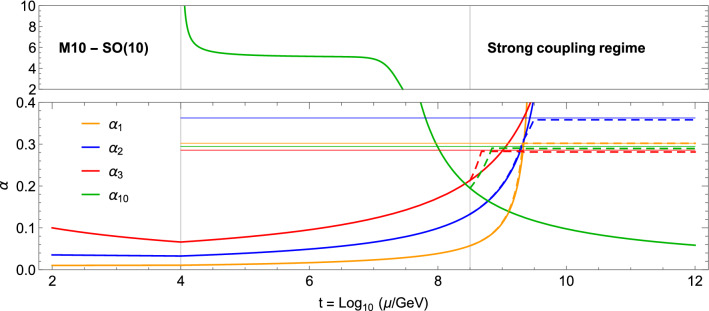
Renormalisation group running of the gauge couplings $\alpha _i$ for model M10, ${\mathcal {G}}_\mathrm{HC} = \text{ SO }(10)$. The dashed lines show the effect of the large-$N_f$ resummation above $\Lambda _\mathrm{Fl} = 10^{8.5}$ GeV. The upper panel shows a cartoon of the running at strong coupling

**Table 2:** Fermion content of the extended M10 (${\mathcal {N}}=10$) and M11 (${\mathcal {N}}=6$) – all fermions are Dirac spinors

	$\text{ SO }({\mathcal {N}})_\mathrm{HC}$	$\text{ SU }(3)_c$	$\text{ SU }(2)_L$	$\text{ U }(1)_Y$	Mass
$\psi _Q$	$\mathbf{Sp}$	1	2	0	$\sim 0$
$\psi _U$	$\mathbf{Sp}$	1	1	1/2	
$\psi _D$	$\mathbf{Sp}$	1	1	$-1/2$	
$\chi _u^3$	$\mathbf{F}$	3	1	2/3	
$\chi _d^3$	$\mathbf{F}$	3	1	$-1/3$	$\sim \Lambda _\mathrm{HC}$
$\chi _l^3$		1	2	$-1/2$	
$\chi _u^{1,2}$	$\mathbf{F}$	3	1	2/3	$$\begin{array}{c} \Lambda _\mathrm{Fl} \\ (\gg \Lambda _\mathrm{HC}) \end{array}$$
$\chi _d^{1,2}$		3	1	$-1/3$	
$\chi _l^{1,2}$		1	2	$-1/2$

**Table 3:** Multiplicity factors for the resummation of the four gauge couplings above $\Lambda _\mathrm{Fl}$. The numerical values in the last two columns refer to M10 (${\mathcal {N}}=10$) and M11 (${\mathcal {N}}=6$)

	$\text{ SO }({\mathcal {N}})_\mathrm{HC}$	M10	M11
$N_{{\mathcal {N}}}$	$24 + 2^{\frac{{\mathcal {N}}-4}{2}}$	32	26
$N_3$	$3 ({\mathcal {N}}+1)$	33	21
$N_2$	$3 + \frac{3}{2} {\mathcal {N}} + 2^{\frac{{\mathcal {N}}-4}{2}}$	26	14
$N_1$	$5 + \frac{13}{2} {\mathcal {N}} + 2^{\frac{{\mathcal {N}}-4}{2}}$	78	46

A key property of the above result is that the function $G_1 (K)$, relevant for non-abelian gauge couplings, has a pole at negative values for $K=3$, while $F_1 (K)$ has a negative pole at $K_1=15/2$, while the resummation fails for coupling values above the pole. This feature, thus, acts as a barrier for the evolution of the respective coupling towards the UV, hinting at the presence of an interacting UV fixed point [10]. In other words, if the value of the coupling at the threshold $\Lambda _\mathrm{Fl}$ is below the pole, the evolution towards the UV will stop at that value where the beta function vanishes and the theory approaches a fixed point. We, therefore, expect the UV fixed point to arise at $K_i = 3$ for non-abelian groups, and $K_1 = 15/2$ for the abelian one. The condition for the model to have a UV safe fixed point for all gauge couplings is that their value is below the pole, i.e.

5 $$\begin{aligned} \alpha _i< \frac{3 \pi }{N_i} \; \text{ for } \; i\ne 1,\quad \text{ and } \; \alpha _1 < \frac{15\pi }{2N_1}. \end{aligned}$$

The above conditions provide an upper bound on $\Lambda _\mathrm{Fl}$ due to the fact that some of the gauge couplings increase towards the UV above $\Lambda _\mathrm{HC}$. On the other hand, an indirect lower bound derives from flavour physics, which gives $\Lambda _\mathrm{Fl} > 10^{5}$ TeV for generic flavour violating effects.

In Fig. 1 we show the running of the 4 gauge couplings above the EW scale for the model M10, assuming $\Lambda _\mathrm{HC} = 10$ TeV (solid lines). While the $\text{ SO }(10)$ gauge coupling $\alpha _{10}$ is asymptotically free, the other three run into a Landau pole below $10^{10}$ GeV. Furthermore, it is the QCD coupling $\alpha _3$ that crosses the UV-safe threshold first, thus setting the maximum allowed value of $\Lambda _\mathrm{Fl}$ right below $10^9$ GeV. In the numerical example, we added the complete set of fermions at a scale $\Lambda _\mathrm{Fl} = 10^{8.5}$ GeV: the modified running is plotted in dashed lines, clearly showing how the gauge couplings approach the UV fixed values. Note that they are a bit below the predicted ones: this is due to the backreaction of the $\text{ U }(1)$ pole on the running of the non-abelian couplings, due to the fact that $F_1 (K_1)$ in Eq. (4) also has a pole at the fixed point. In the upper panel we illustrate the running of $\alpha _{10}$ in the strong coupling regime, which features a walking region between $10^4$ and $10^7$ GeV. This part of the plot, being non-perturbative, can only be confirmed by lattice calculations along the lines of Refs. [34, 39–41]. These results show that the model M10 allows for a narrow mass window where the fermions at $\Lambda _\mathrm{Fl}$ can be added, squeezed between the flavour bounds and the limit from UV safety.

A similar analysis can be done for the model M11, based on ${\mathcal {G}}_\mathrm{HC} = \text{ SO }(6)$: in this case, it is the $\text{ U }(1)$ coupling $\alpha _1$ that crosses the threshold first at a scale around $10^{13}$ GeV, while the $\alpha _2$ and $\alpha _3$ run much slower. This model can therefore allow for a larger flavour scale, and a wider walking window for $\alpha _6$.

The models we have studied here, however, are not truly UV complete, because the dynamics generating the partial compositeness four-fermion interactions is not included. In the following sections we will discuss how scalar mediators can do the job.

## High-scale scalar mediation, and the $\text{ U }(1)$ problem

**Table 4:** Scalar mediators for lepton and quark partial compositeness

	$\text{ SO }({\mathcal {N}})_\mathrm{HC}$	$\text{ SU }(3)_c$	$\text{ SU }(2)_L$	$\text{ U }(1)_Y$	Mass
$\phi _{q}^a$	$\mathbf{Sp}$	3	1	1/6	$\sim \Lambda _\mathrm{Fl}$
$\bar{\phi }_{q}^a$	$\overline{\mathbf{Sp}}$				
$\phi _{l}^a$	$\mathbf{Sp}$	1	1	$-1/2$	$\sim \Lambda _\mathrm{Fl}$
$\bar{\phi }_{l}^a$	$\overline{\mathbf{Sp}}$				

The four-fermion interactions responsible for the partial compositeness couplings at low energy can be generated via scalar mediation. This is acceptable in this class of models because scalar masses are “natural” if they are close to the largest fermion mass in the model [28], namely $m_\phi \approx \Lambda _\mathrm{Fl}$. The only additional condition would be to check that all new couplings in the scalar sector, i.e. Yukawas and quartic couplings, also run to a UV safe fixed point. In this and in the next sections we will address this question.

Firstly, in order to preserve the Dark ${\mathbb {Z}}_2$, it is necessary to add pairs of scalar fields that have the same quantum numbers under the SM gauge symmetries, while they are in conjugate $\mathbf{Sp}$ and $\overline{\mathbf{Sp}}$ *irreps* of the strong $\text{ SO }({\mathcal {N}})$. One minimal set of mediators is shown in Table 4, where $a=1,2,3$ is an index running over the SM generations. These four fields allow to add the following Yukawa couplings above $\Lambda _\mathrm{Fl}$:

6 $$\begin{aligned} {\mathcal {L}}_\mathrm{Yuk, q}= & {} \left\{ \lambda _q^{ab}\ \phi _q^{b,*} [q_L^a]_l [\psi _Q]_l + \lambda _u^{ab} \phi _q^{b,*} [u_R^a]_r [\psi _D]_r \right. \nonumber \\&+ \lambda _d^{ab} \phi _q^{b,*} [d_R^a]_r [\psi _U]_r \nonumber \\&+ \xi _{ur}^{ab}\ \phi _q^a [\chi _u^b]_l^c [\psi _U]_r + \xi _{ul}^{ab}\ \phi _q^a [\chi _u^b]_r^c [\psi _U]_l \nonumber \\&+ \xi _{dr}^{ab}\ \phi _q^a [\chi _d^b]_l^c [\psi _D]_r + \xi _{dl}^{ab}\ \phi _q^a [\chi _d^b]_r^c [\psi _D]_l \nonumber \\&+ \bar{\lambda }_q^{ab}\ \overline{\phi }_q^{b,*} [q_L^a]_l [\psi _Q]_r^c + \bar{\lambda }_u^{ab} \overline{\phi }_q^{b,*} [u_R^a]_r [\psi _U]_l^c \nonumber \\&+ \bar{\lambda }_d^{ab} \overline{\phi }_q^{b,*} [d_R^a]_r [\psi _D]_l^c \nonumber \\&+ \bar{\xi }_{ur}^{ab}\ \overline{\phi }_q^a [\chi _u^b]_l^c [\psi _D]_l^c + \bar{\xi }_{ul}^{ab}\ \overline{\phi }_q^a [\chi _u^b]_r^c [\psi _D]_r^c \nonumber \\&\left. + \bar{\xi }_{dr}^{ab}\ \overline{\phi }_q^a [\chi _d^b]_l^c [\psi _U]_l^c + \bar{\xi }_{dl}^{ab}\ \overline{\phi }_q^a [\chi _d^b]_r^c [\psi _U]_r^c \right\} {\scriptstyle +h.c.},\nonumber \\ \end{aligned}$$

and

7 $$\begin{aligned} {\mathcal {L}}_\mathrm{Yuk, l}= & {} \left\{ \lambda _l^{ab}\ \phi _l^{b,*} [l_L^a]_l [\psi _Q]_l + \lambda _e^{ab} \phi _l^{b,*} [e_R^a]_r [\psi _U]_r \right. \nonumber \\&+ \lambda _\nu ^{ab} \phi _l^{b,*} [\nu _R^a]_r [\psi _D]_r \nonumber \\&+ \xi _{er}^{ab}\ \phi _l^a [\chi _l^b]_l^c [\psi _Q]_r + \xi _{el}^{ab}\ \phi _l^a [\chi _l^b]_r^c [\psi _Q]_l \nonumber \\&+ \bar{\lambda }_l^{ab}\ \overline{\phi }_l^{b,*} [l_L^a]_l [\psi _Q]_r^c + \bar{\lambda }_e^{ab} \overline{\phi }_l^{b,*} [e_R^a]_r [\psi _D]_l^c \nonumber \\&+ \bar{\lambda }_\nu ^{ab} \overline{\phi }_l^{b,*} [\nu _R^a]_r [\psi _U]_l^c \nonumber \\&\left. + \bar{\xi }_{er}^{ab}\ \overline{\phi }_l^a [\chi _l^b]_l^c [\psi _Q]_l^c + \bar{\xi }_{el}^{ab}\ \overline{\phi }_l^a [\chi _l^b]_r^c [\psi _Q]_r^c \right\} {\scriptstyle +h.c.};\nonumber \\ \end{aligned}$$

where a sum over the SM flavour indices is left understood, the subscripts $[.]_{l/r}$ indicate respectively the left and right-handed chiralities, and the superscript $[.]^c$ the charge-conjugation. The Dark parity is preserved as long as $\lambda = \bar{\lambda }$ and $\xi = \bar{\xi }$: as we will see this condition is renormalisation evolution invariant, thus it is preserved at all scales once it is imposed at $\mu = \Lambda _\mathrm{Fl}$. We also need to impose that the two scalars have the same mass. Once they are integrated out, they generate appropriate four-fermion interactions for all SM fermions. As an example, for the top (up-type quarks), among others:

8 $$\begin{aligned} {\mathcal {L}}_{\Lambda _\mathrm{Fl}}= & {} \frac{\kappa _q^{ab}}{\Lambda _\mathrm{Fl}^2} \Big ( [q^a_L]_l \cdot [\psi _Q]_l\ [\chi _u^b]_l^c \cdot [\psi _U]_r \nonumber \\&+ [q_L^a]_l \cdot [\psi _Q]_r^c \ [\chi _u^b]_l^c \cdot [\psi _D]_l^c \Big ) \nonumber \\&+\frac{\kappa _u^{ab}}{\Lambda _\mathrm{Fl}^2} \Big ( [u^a_R]_r \cdot [\psi _D]_r\ [\chi _u^b]_r^c \cdot [\psi _U]_l \nonumber \\&+[u_R^a]_r \cdot [\psi _U]_l^c \ [\chi _u^b]_r^c \cdot [\psi _D]_r^c \Big ), \end{aligned}$$

with

9 $$\begin{aligned} \frac{\kappa _q^{ab}}{\Lambda _\mathrm{Fl}^{2}}= & {} \lambda _q^{ac} [m_\phi ^{2}]^{-1}_{cd} { \xi _{u_l}^{db}}, \nonumber \\ \quad \frac{\kappa _u^{ab}}{\Lambda _\mathrm{Fl}^{2}}= & {} \lambda _u^{ab} [m_\phi ^{2}]^{-1}_{cd} {\xi _{u_r}^{db}}. \end{aligned}$$

The mediators and Yukawa couplings have been selected such that the four-fermion interactions in Eq. (8) generate a coupling of the top fields to a composite baryon in the *irrep* $(\mathbf{4}, \mathbf{4}) \oplus (\bar{\mathbf{4}}, \bar{\mathbf{4}})$ of the global symmetry $\text{ SU }(4)_L \times SU(4)_R$, which we will use in Sect. 6 for the Dark Matter study.

The contribution of the scalars above $\Lambda _\mathrm{Fl}$ will not affect significantly the running of the gauge couplings. The running of the Yukawa couplings above $\Lambda _\mathrm{Fl}$ follows the calculations done in Ref. [12]: it has been observed that the dominant contribution is due to the $\text{ U }(1)$ gauge coupling once it has approached its fixed point, as the contribution to the Yukawa beta function has a pole at the same position. For all Yukawas $y_i$, the beta function can, therefore, be approximated by

10 $$\begin{aligned} \beta (y_i) \approx - y_i \frac{15}{16 \pi ^{2} N_1} (2 d_{y_i,1} + 15 d'_{y_i,1}) \frac{1}{\frac{15}{2} - K_1}, \end{aligned}$$

where $K_1$ is exponentially close (from below) to 15/2, and

11 $$\begin{aligned} d_{y_i,1} = Y_\phi ^{2} + 2 Y_{f1} Y_{f2}, \quad d'_{y_i,1} = \frac{(Y_{f1}-Y_{f2})^{2}}{6}, \end{aligned}$$

with $Y_x$ being the hypercharges of the scalar and fermions in the Yukawa coupling $y_i$. Thus, if

12 $$\begin{aligned} X_{y_i} = 2 d_{y_i,1} + 15 d'_{y_i,1} > 0, \end{aligned}$$

the Yukawa coupling $y_i$ runs to zero in the UV. In our model, we find the values in Table 5, which show that all the Yukawa couplings in Eqs. (6) and (7) are asymptotically free.

**Table 5:** Beta function coefficients for the Yukawas in Eqs. (6) and (7)

$y_i$	$d_{y_i,1}$	$d'_{y_i,1}$	$X_{y_i}$
$y_i$	$d_{y_i,1}$	$d'_{y_i,1}$	$X_{y_i}$
$$\begin{array}{c} \lambda _u,\ \xi _{ur},\ \xi _{ul}, \\ \bar{\lambda }_u,\ \bar{\xi }_{ur},\ \bar{\xi }_{ul} \end{array}$$	$-23/36$	49/216	17/16
$$\begin{array}{c} \lambda _d,\ \xi _{dr},\ \xi _{dl}, \\ \bar{\lambda }_d,\ \bar{\xi }_{dr},\ \bar{\xi }_{dl} \end{array}$$	$-11/36$	25/216	9/16
$\lambda _l,\ \lambda _\nu ,\ \bar{\lambda }_l,\ \bar{\lambda }_\nu$	1/4	1/24	9/16
$$\begin{array}{c} \lambda _e,\ \xi _{er},\ \xi _{el}, \\ \bar{\lambda }_e,\ \bar{\xi }_{er},\ \bar{\xi }_{el} \end{array}$$	$-3/4$	3/8	33/16

While the $\text{ U }(1)$ fixed point drives the Yukawas to be asymptotically free, it is well established that it has a dangerous effect on scalar quartic couplings, which are driven to a Landau pole [12]. We can address this issue by partly unifying the $\text{ U }(1)$ into a non Abelian gauge group, and our model offers an elegant path via a Pati–Salam structure, as discussed in the next section.

## Pati–Salam UV-safe completion

**Fig. 2:**
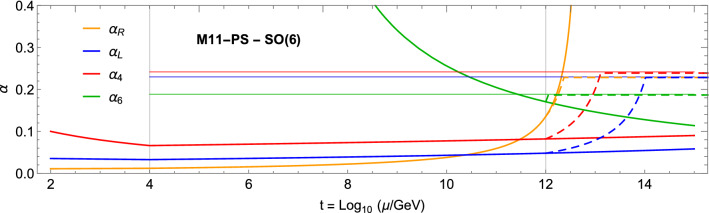
Renormalisation group running of the gauge couplings $\alpha _i$ for model M11-PS, ${\mathcal {G}}_\mathrm{HC} = \text{ SO }(6)$. The dashed lines show the effect of the large-$N_f$ resummation above $\Lambda _\mathrm{Fl} = 10^{12}$ GeV

**Table 6:** Fermion content of the Pati–Salam extended M10-PS (${\mathcal {N}}=10$) and M11-PS (${\mathcal {N}}=6$) – all fermions $\omega$, $\Psi$ and $\Xi$ are Dirac spinors

	$\text{ SO }({\mathcal {N}})_\mathrm{HC}$	$\text{ SU }(4)$	$\text{ SU }(2)_L$	$\text{ SU }(2)_R$	
$\omega _L^a$	$\mathbf{1}$	4	2	1	$q_L^a,\ l_L^a$
$\omega _R^a$	$\mathbf{1}$	4	1	2	$$\begin{array}{c} u_R^a,\ d_R^a,\\ e_L^a,\ \nu _R^a \end{array}$$
$\Psi _{L}$	$\mathbf{Sp}$	1	2	1	$\psi _Q$
$\Psi _R$	$\mathbf{Sp}$	1	1	2	$\psi _U,\ \psi _D$
$\Xi _R^a$	$\mathbf{F}$	4	1	2	$\chi _u^a,\ \chi _d^a$
$\Xi _L^a$	$\mathbf{F}$	4	2	1	$\chi _l^a$
$\Phi ^a$	$\mathbf{Sp}$	4	1	1	$\phi _q^a,\ \phi _l^a$
$\overline{\Phi }^a$	$\overline{\mathbf{Sp}}$	4	1	1	$\overline{\phi }_q^a,\ \overline{\phi }_l^a$
$\varphi _\mathrm{PS}$	$\mathbf{1}$	4	1	2	−

**Table 7:** Multiplicity factors for the gauge couplings in the Pati–Salam UV completions

	$\text{ SO }({\mathcal {N}})_\mathrm{HC}$	M10-PS	M11-PS
$N_{{\mathcal {N}}}$	$48 + 2^{\frac{{\mathcal {N}}-4}{2}}$	56	50
$N_4$	$3 (2 {\mathcal {N}}+1)$	63	39
$N_L = N_R$	$3 + 6 {\mathcal {N}} + 2^{\frac{{\mathcal {N}}-4}{2}}$	71	41

To remove the destabilising effect of the $\text{ U }(1)$ pole on scalar quartic couplings, we can embed the model above $\Lambda _\mathrm{Fl}$ into a Pati–Salam [42] partial unification for the SM interactions [43]. The new models, that we dub M10-PS and M11-PS, feature the field content in Table 6. The Pati–Salam gauge group, $\text{ SU }(4) \times SU(2)_L \times \text{ SU }(2)_R$ is broken by a scalar field $\varphi _\mathrm{PS}$, with a vacuum expectation value of the order of $\Lambda _\mathrm{Fl}$. To study the UV properties of this model, we can calculate the beta functions following the same procedure highlighted in Sect. 3, with the important difference that the contribution of other gauge couplings remains negligible due to the absence of an abelian group. The new $N_i$ are given in Table 7: they are substantially larger than the corresponding ones in the previous models, indicating lower values for the UV fixed points. This is potentially dangerous, as the upper limit on $\Lambda _\mathrm{Fl}$ will tend to decrease.

In Fig. 2 we show the running of the Pati–Salam gauge couplings in the model M11-PS^4^: the first coupling to pass the safe threshold is $\alpha _R$, at an energy scale slightly lower than that for M11. In the numerical example, we fixed $\Lambda _\mathrm{Fl} = 10^{12}$ GeV, where the field content of M11-PS is added. Besides the difference in scales, the approach to the UV fixed point is similar, also showing the same fixed point for the two $\text{ SU }(2)$’s thanks to the left-right symmetry of the model. For M10-PS, we find that the maximum allowed value for $\Lambda _\mathrm{Fl}$ is slightly above $10^{7}$ GeV, thus generating potential conflict with flavour bounds and also leaving too small space for the IR walking window. These results show that M11-PS is favoured.

We can now study the safety of the Yukawa couplings, which can be written in the Pati–Salam unified models as

13 $$\begin{aligned} {\mathcal {L}}_\mathrm{Yuk, PS}= & {} \left\{ \gamma _L^{ab}\ \Phi ^{b,*} [\omega _L^a]_l [\Psi _L]_l + \gamma _R^{ab} \Phi ^{b,*} [\omega _R]_r [\Psi _R]_r \right. \nonumber \\&+ \zeta _{Rr}^{ab}\ \Phi ^a [\Xi _R^b]_l^c [\Psi _R]_r + \zeta _{Rl}^{ab}\ \Phi ^a [\Xi _R^b]_r^c [\Psi _R]_l \nonumber \\&+ \zeta _{Lr}^{ab}\ \Phi ^a [\Xi _L^b]_l^c [\Psi _L]_r + \zeta _{Ll}^{ab}\ \Phi ^a [\Xi _L^b]_r^c [\Psi _L]_l \nonumber \\&+ \bar{\gamma }_L^{ab}\ \overline{\Phi }^{b,*} [\omega _L^a]_l [\Psi _L]_r^c + \bar{\gamma }_R^{ab} \overline{\Phi }^{b,*} [\omega _R]_r [\Psi _R]_l^c \nonumber \\&+ \bar{\zeta }_{Rr}^{ab}\ \overline{\Phi }^a [\Xi _R^b]_l^c [\Psi _R]_l^c + \bar{\zeta }_{Rl}^{ab}\ \overline{\Phi }^a [\Xi _R^b]_r^c [\Psi _R]_r^c \nonumber \\&\left. + \bar{\zeta }_{Lr}^{ab}\ \overline{\Phi }^a [\Xi _L^b]_l^c [\Psi _L]_l^c + \bar{\zeta }_{Ll}^{ab}\ \overline{\Phi }^a [\Xi _L^b]_r^c [\Psi _L]_r^c \right\} \nonumber \\&{\scriptstyle +h.c.}. \end{aligned}$$

In absence of $\text{ U }(1)$ couplings, the contribution of Yukawas and gauge couplings can be comparable. The beta function can be written as [12, 38]

14 $$\begin{aligned} (\beta _y)_{aij}= & {} \frac{1}{32 \pi ^{2}} \Big \{ (y_b \cdot y^{\dagger ,b}\cdot y_a)_{ij} + (y_a \cdot y^{\dagger ,b} \cdot y_b)_{ij} \nonumber \\&+ 2 \text{ Tr } [y_a \cdot y^{\dagger ,b}] y_{bij} \Big \}\nonumber \\&- \frac{3}{2} y_{aij} \sum _\alpha \frac{K_\alpha }{N_\alpha } H_0 (K_\alpha ) \nonumber \\&\times \left( \frac{C_2 (f_1) + C_2 (f_2)}{2} + \frac{C_2 (\Phi )}{12} K_\alpha \right) , \end{aligned}$$

where $\alpha$ indicates the sum over the 4 gauge groups, and $C_2$ are the Casimirs of the *irreps* of the two fermions and scalar under each gauge group. The function $H_0$ remains finite up to $K < 15/2$, thus the gauge contribution remains small up to the UV fixed points, reached for $K_\alpha = 3$ (where $H_0 (3) = 1/9$). The beta function, after the gauge couplings have reached the fixed points, thus reads

15 $$\begin{aligned} \beta (y_k) = \frac{y_k}{32 \pi ^{2}} \sum _{p} d_{kp} y_p^{2} - C_{y_k} y_k, \end{aligned}$$

where $d_{kp}>0$ and order 1. Thus all Yukawas run to zero as long as $C_{y_k} > 0$ and $y_k$ is small enough at $\Lambda _\mathrm{Fl}$ that the beta functions are negative. We find that the $C_{y_k}$ are the same for all the $\gamma$-type ($\bar{\gamma }$) and $\zeta$-type ($\bar{\zeta }$) Yukawas in Eq. (13), with

16 $$\begin{aligned} \begin{array}{c} C_\gamma = 0.046, C_\zeta = 0.066, \text{ for } \text{ M10-PS }; \\ C_\gamma = 0.028, C_\zeta = 0.040, \text{ for } \text{ M11-PS }. \end{array} \end{aligned}$$

As they are all positive and one order of magnitude larger than the factor $\frac{1}{32 \pi ^{2}}$, the Yukawas are asymptotically free in both models with values of ${\mathcal {O}} (1)$ allowed at $\Lambda _\mathrm{Fl}$.

## Dark matter phenomenology

**Fig. 3:**
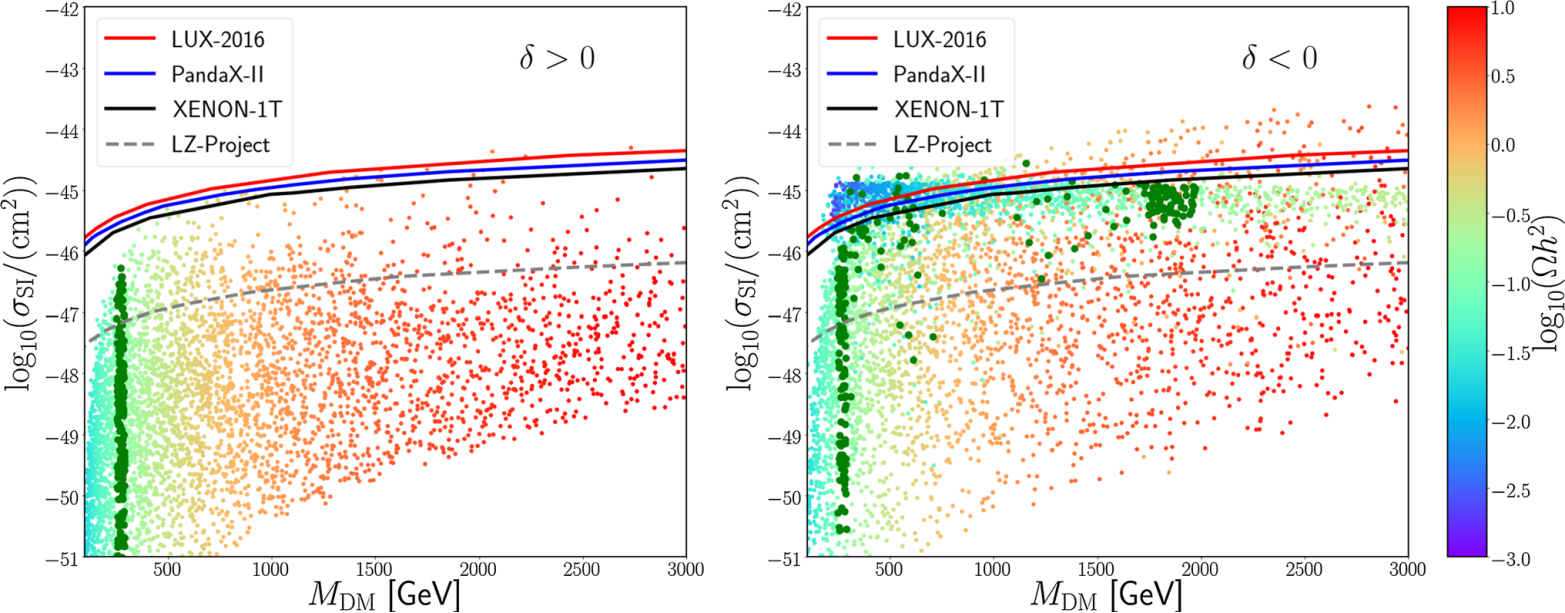
Direct detection constraints [44–46] for $\delta > 0$ (left) and $\delta < 0$ (right). The colour encodes the relic density for each parameter point, which is used to rescale the spin-independent cross section $\sigma _\mathrm{SI}$. The dark green points saturate the measured relic density value, with points having warmer colour being excluded by over-density

The low energy physics of the two models, M10 and M11, can be described by the same effective field theory as they have the same global symmetries. The only distinction, besides the value of the low energy constants, can be traced in the properties of the pNGBs associated to the global $\text{ U }(1)$ symmetries broken by the condensates [32, 47]. The Dark Matter sector is similar to that of the model in Ref. [37]: the odd pNGBs consist of a triplet of $\text{ SU }(2)_L$, an inert Higgs doublet and charged and neutral singlets (forming a triplet of the custodial $\text{ SU }(2)_R$). However, the pNGB potential generated by the top interactions is very different, as here we use partial compositeness to generate the top mass, while in Ref. [37] bilinear Yukawa-like interactions are considered. Thus, mass spectra and couplings are different from those in the model of Ref. [37]. In this work we explicitly computed loops of the top and top partners after imposing the maximal symmetry [48] to keep the loops calculable and finite.

The nature of the lightest neutral stable scalar crucially depends on the masses of the preons: here we assume that the $\psi _U$ and $\psi _D$ have a common mass $m_R$ in order to preserve the global custodial $\text{ SU }(2)_R$, while the mass of $\psi _Q$ is $m_L$. The crucial parameter is thus the mass difference $\delta \equiv (m_{L}-m_{R})/(m_{L}+m_{R})$. For $\delta < 0$, the lightest neutral stable scalar mostly coincides with the $\text{ SU }(2)_L$ triplet, while for $\delta > 0$ it has maximal overlap with the singlets. For $\delta \sim 0$, maximal mixing with the doublet is active. This mixing pattern determines the annihilation rates of the Dark Matter candidate, which is dominated by the final states in two EW gauge bosons and two tops. The annihilation cross section is thus larger for $\delta < 0$, leading to larger allowed dark matter masses.

To study a concrete example, we computed the top and gauge boson loop potential, considering case a), where the top partners transform in the anti-symmetric of the unbroken $\text{ SU }(4)$, as embedded in the bi-fundamental (see 1 for more details). We constrain the parameter space by fixing the masses of the top (173 GeV) and Higgs (125 GeV) at the minimum of the potential. We then scan the remaining parameter space and compute relic abundance and spin-independent cross section off nuclei by using the micrOMEGAs [49] package. For the misalignment angle, $\sin \theta = \frac{v}{f}$, we probe values between 0.0003 and 0.3. Here, $v = 246$ GeV is the SM Higgs vacuum expectation value, $f \sim \frac{\Lambda _\mathrm{HC}}{4\pi }$ the decay constant of the composite Higgs, and the Dark Matter mass is proportional to *f*. In Fig. 3 we show the results of our scan in the plane of the dark matter mass versus the cross section rescaled by the actual relic abundance. In this way, all points can be compared to the direct detection exclusion, shown by the solid black, blue and red lines. Points that saturate the relic abundance within 10$\sigma$ are highlighted in dark green. We see that the model can explain the Dark Matter abundance without being excluded for $M_\mathrm{DM} \approx 260$ GeV, while larger values up to 1.5–2 TeV are allowed for $\delta < 0$. The region at DM masses close to 260 GeV is dominated by co-annihilation with the next-to-lightest odd state ($\phi _1^\pm$) due to the small mass splitting. The fixed value of the DM mass stems from the dominance of the annihilation process $\phi _1^+ \phi _1^- \rightarrow \gamma \gamma$ via gauge interactions: thus, the relic density highly depends on the masses. Note also that the larger Dark Matter masses will be probed by future direct detection experiments. We remark that those masses have reasonable values compared to the typical compositeness scale at the TeV. While in the $\delta > 0$ case, being the DM dominantly a singlet, indirect detection constraints can be neglected, in the $\delta < 0$ case they might be relevant [30, 50]. However, we have checked that the current bounds are very weak and not competitive with direct detection.

We also performed a similar scan for top partners in case b), finding that all points saturating the relic density are excluded by direct detection. Having demonstrated that a feasible Dark Matter candidate is present in the model, we leave a detailed study of the low energy phenomenology of these models for future work.

## Conclusions and outlook

We have presented a new paradigm that allows to define composite Higgs models with partial compositeness for the top quark up to arbitrarily high scales. For the first time, we can endow composite models with predictivity power. Based on gauge-fermion underlying descriptions of the low energy physics, we use the need for a large multiplicity of fermions, related to the large number of fermions and generations in the Standard Model, to predict the presence of UV safe fixed points for the complete theory.

We apply this paradigm to models that also feature a composite scalar Dark Matter candidate. We show that the gauge couplings, which include the coupling of the confining group $\text{ SO }(10)_\mathrm{HC}$ (for M10) or $\text{ SO }(6)_\mathrm{HC}$ (for M11), can develop a UV interacting fixed point while also allowing for an IR conformal window and a sufficient hierarchy between the scale of flavour physics generation and the EW scale. Furthermore, a Dark Matter candidate is predicted in a consistent mass ballpark, which can also saturate the relic abundance while evading direct detection bounds.

In the paradigm we propose, the four fermion interactions corresponding to partial compositeness for the Standard Model fermions are generated by scalar mediators with a mass close to $\Lambda _\mathrm{Fl}$, i.e. the scale where the theory approaches the UV fixed point. We showed that the Yukawa couplings run to zero in the UV, thus not spoiling the safety of the model. The well known instability on the scalar quartic couplings can be cured by embedding the model in a Pati–Salam envelope above $\Lambda _\mathrm{Fl}$. We have shown that the two models, M10-PS and M11-PS, also feature an interacting fixed point for the gauge couplings, and asymptotically free Yukawas, while M11-PS based on $\text{ SO }(6)_\mathrm{HC}$ is preferred due to the higher flavour scale. This also leaves open the possibility that the four-fermion interactions are generated by vector mediators, à *la* Extended Technicolor. We leave the investigation of this point for further work.

The results presented in this work are stepping stones towards complete composite Higgs models, where the origin of the standard fermion masses can be finally addressed. Some crucial ingredients, like the presence of an IR window where large anomalous dimensions are generated, need input from lattice calculations, possible as a detailed underlying model is on the table. The collider phenomenology of composite models is also affected, as non-minimal cosets are the norm in this scenario, thus predicting additional charged and neutral light scalars that can be searched for at the LHC.

## Data Availability

This manuscript has no associated data or the data will not be deposited. [Authors’ comment: This is a theoretical study and has no experimental data associated to it.]

## Appendix A: Conformal window

To estimate if the model below $\Lambda _\mathrm{Fl}$ is inside the IR conformal window, we will utilise the Schwinger–Dyson rainbow approximation [51, 52] and the beta-function at two loops to estimate the position of the fixed point. Following Ref. [53], the beta functions read:

A.1 $$\begin{aligned} \beta _0= & {} \frac{11}{3} C_2(\mathbf{G}) - \frac{4}{3} \sum _{i=\psi , \chi } T(r_i) n_i , \nonumber \\ \beta _1= & {} \frac{34}{3} C_2^{2} (\mathbf{G}) \nonumber \\&- \frac{4}{3} \sum _{i=\psi , \chi } \left[ 5 C_2 (\mathbf{G}) + 3 C_2 (r_i) \right] T(r_i) n_i, \end{aligned}$$

where $\mathbf{G}$ indicates the adjoint representation. The IR fixed point, if existent, is characterised by the gauge coupling:

A.2 $$\begin{aligned} \alpha ^{*} = - 4 \pi \frac{\beta _0}{\beta _1}, \end{aligned}$$

assuming that $\beta _1 < 0$ (and $\beta _0 >0$ as the theory is asymptotically free). In the rainbow approximation, we can calculate the value of the gauge coupling where the condensate of the two species become critical: as the theory flows from an UV free point, the critical value is given by the smallest value of the two condensates:

A.3 $$\begin{aligned} \alpha _c = \text{ min } \left\{ \frac{\pi }{3 C_2 (r_i)} \right\} . \end{aligned}$$

Equating $\alpha _c \equiv \alpha ^{*}$ determines the lower edge of the conformal window. For the two models M10 and M11, fixing $n_\psi = 4$, we can find the range of $n_\chi$ leading to a theory inside the IR conformal window:

A.4 $$\begin{aligned} \text{ SO }(10)\Rightarrow & {} 4< n_\chi < 14, \end{aligned}$$

A.5 $$\begin{aligned} \text{ SO }(6)\Rightarrow & {} 6< n_\chi < 9, \end{aligned}$$

where the upper edge is determined by the loss of asymptotic freedom. The models in Table 2 have $n_\chi = 8$ below $\Lambda _\mathrm{Fl}$, thus they are expected to be well inside the conformal window.

As large anomalous dimensions are needed to enhance the top partial compositeness in particular, it may be needed to push the theory closer to the lower edge, where the coupling is stronger. In M10, for instance, one could push the mass of $\chi _d^3$ close to $\Lambda _\mathrm{Fl}$ in order to have $n_\chi = 5$ (while the bottom mass could be generated by the chimera baryons containing $\chi _u^3$). For M11, one could replace $\chi _l^3$ with an $\text{ SU }(2)_L$ singlet with hypercharge $-1$, thus leading to $n_\chi = 7$: this will marginally affect the running above $\Lambda _\mathrm{Fl}$, while only two neutrino masses can be generated (i.e., predicting one massless neutrino).

An alternative method to determine the conformal window, proposed in Ref. [54], is based on an all-order beta function conjecture, and it would lead to a lower edge for the conformal window, leading to $n_\chi > 3$ for both models.

## Appendix B: Resummation

The functions appearing in the gauge coupling running are defined as [38]:

B.6 $$\begin{aligned} G_1 (K)= & {} \frac{3}{4} \int _0^K dx\ \tilde{F} \left( 0,\frac{2}{3} x\right) \ \tilde{g} \left( \frac{1}{3} x\right) , \nonumber \\ F_1 (K)= & {} \frac{3}{4} \int _0^K dx\ \tilde{F} \left( 0,\frac{2}{3} x\right) , \end{aligned}$$

where

$$\begin{aligned} \tilde{F} (0,y)= & {} \frac{(1-y) (1-\frac{y}{3}) (1+\frac{y}{2}) \Gamma (4-y)}{3 \Gamma ^{2} (2-\frac{y}{2}) \Gamma (3-\frac{y}{2}) \Gamma (1+ \frac{y}{2})}, \\ \tilde{g} (y)= & {} \frac{20-43 y + 32 y^{2} - 14 y^3 + 4 y^4}{4 (2y-1)(2y-3)(1-y^{2})}. \end{aligned}$$

We remark that the pole in $K=15/2$ comes from the $\Gamma (4-y)$–factor in $\tilde{F}$, which diverges for $y \rightarrow 5$, while the pole in $K=3$ for $G_1 (K)$ comes from the factor $(1-y^{2})$ at the denominator of $\tilde{g}$. We remind the reader that $F_1 (K)$ corresponds to the resummation for abelian gauge groups [8], and it encodes 2-loop diagrams with fermion bubbles inserted in the gauge propagators. On the other hand, $G_1 (K)$ includes the contribution of 2-loop diagrams involving the triliner gauge boson self coupling [9, 55], with fermion bubble insertions. It is useful to connect our definitions with the function $H_1$ defined in Ref. [12]:

B.7 $$\begin{aligned} H_1 = \frac{C_2 (\mathbf{G})}{C_2 (R_f)} \left( -\frac{11}{4} + G_1 \right) + F_1. \end{aligned}$$

For the UV completions of the models M10 and M11, we find:

B.8 $$\begin{aligned} c_{{\mathcal {N}},{\mathcal {N}}}= & {} C_2 (\mathbf{F}) = \frac{{\mathcal {N}}-1}{2}, \end{aligned}$$

B.9 $$\begin{aligned} c_{{\mathcal {N}},3}= & {} 24 \frac{T(\mathbf{F})}{N_{\mathcal {N}}}, \end{aligned}$$

B.10 $$\begin{aligned} c_{{\mathcal {N}},2}= & {} \frac{3 T(\mathbf{F})}{2 N_{{\mathcal {N}}}} \left( 3 + \frac{T (\mathbf{Sp})}{T(\mathbf{F})} \right) , \end{aligned}$$

B.11 $$\begin{aligned} c_{{\mathcal {N}},1}= & {} \frac{T(\mathbf{F})}{N_{{\mathcal {N}}}} \left( \frac{13}{2} + \frac{T (\mathbf{Sp})}{2 T(\mathbf{F})} \right) , \end{aligned}$$

B.12 $$\begin{aligned} c_{3, {\mathcal {N}}}= & {} 3 \frac{T(\mathbf{F}) d(\mathbf{G})}{N_3}, \end{aligned}$$

B.13 $$\begin{aligned} c_{3,3}= & {} \frac{4}{3}, \end{aligned}$$

B.14 $$\begin{aligned} c_{3,2}= & {} \frac{9}{8 N_3}, \end{aligned}$$

B.15 $$\begin{aligned} c_{3,1}= & {} \frac{1}{N_3} \left( \frac{5}{6} d (\mathbf{F}) + \frac{11}{24} \right) , \end{aligned}$$

B.16 $$\begin{aligned} c_{2,{\mathcal {N}}}= & {} \frac{T(\mathbf{F}) d (\mathbf{G})}{N_2} \left( \frac{3}{2} + \frac{T(\mathbf{Sp})}{2 T(\mathbf{F})} \right) , \end{aligned}$$

B.17 $$\begin{aligned} c_{2,3}= & {} \frac{3}{N_2}, \end{aligned}$$

B.18 $$\begin{aligned} c_{2,2}= & {} \frac{3}{4}, \end{aligned}$$

B.19 $$\begin{aligned} c_{2,1}= & {} \frac{1}{N_2} \left( \frac{3}{8} d (\mathbf{F}) + \frac{1}{4} \right) , \end{aligned}$$

B.20 $$\begin{aligned} c_{1,{\mathcal {N}}}= & {} \frac{T(\mathbf{F}) d(\mathbf{G})}{N_1} \left( \frac{13}{2} + \frac{T(\mathbf{Sp})}{2 T(\mathbf{F})} \right) , \end{aligned}$$

B.21 $$\begin{aligned} c_{1,3}= & {} \frac{1}{N_1} \left( \frac{20}{3} d(\mathbf{F}) + \frac{11}{3} \right) , \end{aligned}$$

B.22 $$\begin{aligned} c_{1,2}= & {} \frac{1}{N_1} \left( \frac{9}{8} d(\mathbf{F}) + \frac{3}{4} \right) , \end{aligned}$$

B.23 $$\begin{aligned} c_{1,1}= & {} \frac{1}{N_1} \left( \frac{163}{72} d(\mathbf{F}) + \frac{1}{8} d(\mathbf{Sp}) + \frac{95}{36} \right) . \end{aligned}$$

The group theory factors appearing in the above expressions refer to the $\text{ SO }({\mathcal {N}})$ *irreps*, and are equal to

$$\begin{aligned} \begin{array}{ccc} d(\mathbf{G}) = \frac{{\mathcal {N}} ({\mathcal {N}}-1)}{2}, &{} d(\mathbf{F}) = {\mathcal {N}}, &{} d(\mathbf{Sp}) = 2^\frac{{\mathcal {N}}-2}{2}, \\ T(\mathbf{G}) = {\mathcal {N}}-2, &{} T(\mathbf{F}) = 1, &{} T(\mathbf{Sp}) = 2^\frac{{\mathcal {N}}-8}{2}, \\ C_2(\mathbf{G}) = {\mathcal {N}}-2, &{} C_2(\mathbf{F}) = \frac{{\mathcal {N}}-1}{2}, &{} C_2(\mathbf{Sp}) = \frac{{\mathcal {N}}({\mathcal {N}}-1)}{16}. \end{array} \end{aligned}$$

Numerically, for M10 we find:

B.24 $$\begin{aligned} c_{i,j}^{M10} = \begin{pmatrix} 4.5 &{} 0.75 &{} 0.234 &{} 0.234 \\ 4.09 &{} 1.33 &{} 0.068 &{} 0.266 \\ 4.33 &{} 0.115 &{} 0.75 &{} 0.154 \\ 4.33 &{} 0.90 &{} 0.154 &{} 0.350 \end{pmatrix}; \end{aligned}$$

while for M11:

B.25 $$\begin{aligned} c_{i,j}^{M11} = \begin{pmatrix} 2.5 &{} 0.923 &{} 0.202 &{} 0.260 \\ 2.14 &{} 1.33 &{} 0.107 &{} 0.260 \\ 1.88 &{} 0.214 &{} 0.75 &{} 0.179 \\ 2.20 &{} 0.949 &{} 0.163 &{} 0.364 \end{pmatrix}; \end{aligned}$$

where $$i,j = {\mathcal {N}},3,2,1$$.

### Appendix C: Top partners

For partial compositeness, based on the UV completions in Table 2, we are considering the case where composite top partners transform in the antisymmetric representations 6 and $\bar{6}$ under unbroken subgroup $\text{ SU }(4)$ of the global symmetry $\text{ SU }(4)_L\times \text{ SU }(4)_R$. In order to preserve the Dark parity in the theory, we shall include both representations in a symmetric way. To write the proper mixing terms, the elementary top quark fields need to be embedded in the above representations, by way of the following spurions:

C.26 $$\begin{aligned} \psi _{q_L}^{u,6}&= \frac{1}{\sqrt{2}}\left( \begin{array}{cccc} 0 &{} 0 &{} 0 &{} t_L \\ 0 &{} 0 &{} 0 &{} b_L \\ 0 &{} 0 &{} 0 &{} 0 \\ -t_L &{} -b_L &{} 0 &{} 0 \end{array}\right) ,\nonumber \\ \psi _{q_L}^{d,6}&= \frac{1}{\sqrt{2}}\left( \begin{array}{cccc} 0 &{} 0 &{} t_L &{} 0 \\ 0 &{} 0 &{} b_L &{} 0 \\ -t_L &{} -b_L &{} 0 &{} 0 \\ 0 &{} 0 &{} 0 &{} 0 \end{array}\right) , \nonumber \\ \psi _{q_L}^{u,\bar{6}}&= \frac{1}{\sqrt{2}}\left( \begin{array}{cccc} 0 &{} 0 &{} -b_L &{} 0 \\ 0 &{} 0 &{} t_L &{} 0 \\ b_L &{} -t_L &{} 0 &{} 0 \\ 0 &{} 0 &{} 0 &{} 0 \end{array}\right) ,\nonumber \\ \psi _{q_L}^{d,\bar{6}}&= \frac{1}{\sqrt{2}}\left( \begin{array}{cccc} 0 &{} 0 &{} 0 &{} b_L \\ 0 &{} 0 &{} 0 &{} -t_L \\ 0 &{} 0 &{} 0 &{} 0 \\ -b_L &{} t_L &{} 0 &{} 0 \end{array}\right) , \nonumber \\ \psi _{t_R}^{6(\bar{6})}&=\frac{1}{\sqrt{2}}\left( \begin{array}{cccc} 0 &{} t_R &{} 0 &{} 0 \\ -t_R &{} 0 &{} 0 &{} 0 \\ 0 &{} 0 &{} 0 &{} 0 \\ 0 &{} 0 &{} 0 &{} 0 \end{array}\right) , \nonumber \\ \psi _{b_R}^{6(\bar{6})}&=\frac{1}{\sqrt{2}}\left( \begin{array}{cccc} 0 &{} b_R &{} 0 &{} 0 \\ -b_R &{} 0 &{} 0 &{} 0 \\ 0 &{} 0 &{} 0 &{} 0 \\ 0 &{} 0 &{} 0 &{} 0 \end{array}\right) . \end{aligned}$$

The transformation properties of these spurions are

C.27 $$\begin{aligned} \psi _{q_L}^{u/d,6}&\rightarrow L \psi _{q_L}^{u/d,6} L^T, \nonumber \\ \psi _{q_L}^{u/d,\bar{6}}&\rightarrow R^{*}\psi _{q_L}^{u/d,\bar{6}} R^{\dagger }, \nonumber \\ \psi _{t_R/b_R}^{6}&\rightarrow L \psi _{t_R/b_R}^{6} L^{T}, \nonumber \\ \psi _{t_R/b_R}^{\bar{6}}&\rightarrow R^{*} \psi _{t_R/b_R}^{\bar{6}} R^{\dagger }, \end{aligned}$$

where *L* (*R*) is an element of the global symmetry $\text{ SU }(4)_L$ ($\text{ SU }(4)_R$).

At low energies, the partial compositeness Lagrangian can be written as

C.28 $$\begin{aligned} {\mathcal {L}}&= \epsilon _L^{u,6}f\mathrm{Tr}[\bar{\psi }_{q_L}^{u,6}U{\mathcal {B}}_{q_L}^{u,6}U^T] + \epsilon _L^{u,\bar{6}}f\mathrm{Tr}[\bar{\psi }_{q_L}^{u,\bar{6}}U^T{\mathcal {B}}_{q_L}^{u,\bar{6}}U] \nonumber \\&\quad + \epsilon _L^{d,6}f\mathrm{Tr}[\bar{\psi }_{q_L}^{d,6}U{\mathcal {B}}_{q_L}^{d,6}U^T] + \epsilon _L^{d,\bar{6}}f\mathrm{Tr}[\bar{\psi }_{q_L}^{d,\bar{6}}U^T{\mathcal {B}}_{q_L}^{d,\bar{6}}U] \nonumber \\&\quad + \epsilon _R^{u,6}f\mathrm{Tr}[\bar{\psi }_{t_R}^{6}U{\mathcal {B}}_{t_R}^{6}U^T]+\epsilon _R^{u,\bar{6}}f\mathrm{Tr}[\bar{\psi }_{t_R}^{\bar{6}}U^T{\mathcal {B}}_{t_R}^{\bar{6}}U] \nonumber \\&\quad + \epsilon _R^{d,6}f\mathrm{Tr}[\bar{\psi }_{b_R}^{6}U{\mathcal {B}}_{b_R}^{6}U^T]+\epsilon _R^{d,\bar{6}}f\mathrm{Tr}[\bar{\psi }_{b_R}^{\bar{6}}U^T{\mathcal {B}}_{b_R}^{\bar{6}}U] \nonumber \\&\quad + M_{6\bar{6}}^{u}\mathrm{Tr}[\bar{{\mathcal {B}}}_{q_L}^{u,6}{\mathcal {B}}_{t_R}^{\bar{6} \prime }] + M_{\bar{6}6}^{u}\mathrm{Tr}[\bar{{\mathcal {B}}}_{q_L}^{u,\bar{6}}{\mathcal {B}}_{t_R}^{6 \prime }] \nonumber \\&\quad + M_{6\bar{6}}^{d}\mathrm{Tr}[\bar{{\mathcal {B}}}_{q_L}^{d,6}{\mathcal {B}}_{b_R}^{\bar{6} \prime }] + M_{\bar{6}6}^{d}\mathrm{Tr}[\bar{{\mathcal {B}}}_{q_L}^{d,\bar{6}}{\mathcal {B}}_{b_R}^{6 \prime }] + h.c. \end{aligned}$$

where the ${\mathcal {B}}$’s are the corresponding top partners, ${\mathcal {B}}^{ \prime }_{ij} =\epsilon _{ij kl} {\mathcal {B}}_{kl}$ and *U* is the usual non-linear sigma field, which transforms under the global symmetry as

C.29 $$\begin{aligned}&U \rightarrow L U h^\dagger , \quad U \rightarrow h U R^{\dagger }, \end{aligned}$$

where *h* is an element of the unbroken group $\text{ SU }(4)$. In order to preserve the Dark parity, the following conditions must be satisfied:

C.30 $$\begin{aligned} \epsilon _L^{u,6}&= -\epsilon _L^{u,\bar{6}} \equiv \epsilon _L^u,\quad \epsilon _L^{d,6} =- \epsilon _L^{d,\bar{6}} \equiv \epsilon _L^d, \nonumber \\ \epsilon _R^{u,6}&= \epsilon _R^{u,\bar{6}} \equiv \epsilon _R^u,\quad \epsilon _R^{d,6} = \epsilon _R^{d,\bar{6}} \equiv \epsilon _R^d, \nonumber \\ M_{6\bar{6}}^{u}&= M_{\bar{6}6}^u \equiv M_\Delta ^u, \quad M_{6\bar{6}}^d = M_{\bar{6}6}^d \equiv M_\Delta ^d. \end{aligned}$$

It is straightforward to verify that the underlying Lagrangian is invariant under the DM parity with the following transformations:

C.31 $$\begin{aligned} \psi _{X}^{6}&\leftrightarrow -P_B ( \psi _{X}^{\bar{6}} ) P_B^{\dagger } =\psi _{X}^{6}, \nonumber \\ {\mathcal {B}}_{X}^{6}&\leftrightarrow P_B {\mathcal {B}}_{X}^{\bar{6}} P_B^{\dagger }, \quad U \rightarrow P_B U^T P_B^{\dagger }; \end{aligned}$$

where $P_B$ is the Dark parity transformation defined in [37]:

$$\begin{aligned} P_B = \left( \begin{array}{cc} \sigma _2 &{} 0 \\ 0 &{} -\sigma _2 \end{array}\right) . \end{aligned}$$

The scalar potential has a similar form to the one used in [29, 37], with the fermion-induced potential replaced by the one induced by loops of the above partial compositeness Lagrangian. The full potential has been coded into FeynRules [56], and then interfaced to micrOMEGAs [57]. The latter tool is then used to calculate the DM relic density as well as the scattering cross-sections for direct and indirect detection.

## Footnotes

1 Reference [15] reported a first attempt to calculate the second term for abelian gauge symmetry.

2 Quantum gravity effects may also be able to drive the Standard Model interactions to a safe UV, see for instance Ref. [21].

3 Note that the protection in this safe model is against modifications of the UV running of gauge couplings due, for instance, to the presence of Landau poles. This is the case for the hypercharge coupling in the SM. We do not address here the corrections coming from the Planck scale, which can only be treated once a consistent theory of gravity is integrated with the model.

4 The usual matching applies:

$$\begin{aligned} \alpha _4 = \alpha _3, \alpha _L = \alpha _2, \alpha _R = \frac{3 \alpha _1 \alpha _3}{3 \alpha _3 - 2 \alpha _1}. \end{aligned}$$
